# Public health round-up

**DOI:** 10.2471/BLT.22.010822

**Published:** 2022-08-01

**Afghanistan earthquake**

A two-year-old girl at a United Nations Children’s Fund-supported mobile health clinic in Gayan District, Paktika Province, Afghanistan, where she was being treated for cholera. As of 28 June 2022, over 1000 people had been killed and over 3600 had been injured as a result of the earthquake that hit Afghanistan on 22 June.

**Figure:**
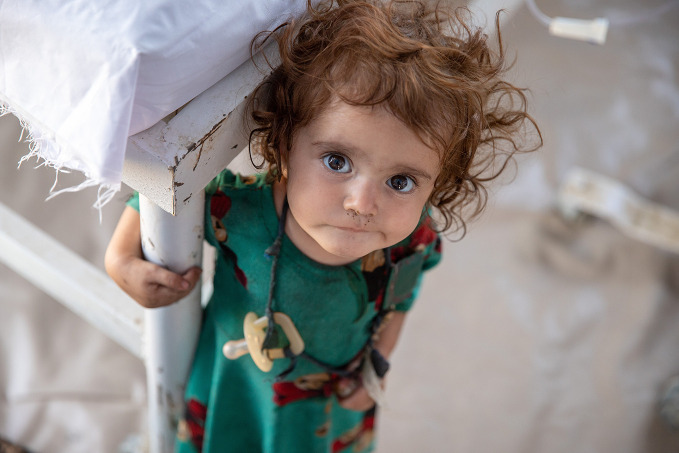
UNICEF/Sayed Bidel

## East Africa nutrition crisis

The eastern Africa region is facing a nutrition crisis driven by conflict, extreme weather events – including the worst drought in 40 years – rising international food and fuel prices and the impact of the coronavirus disease 2019 (COVID-19) pandemic. According to a World Health Organization (WHO) statement issued on 30 June, seven countries are affected (Djibouti, Ethiopia, Kenya, Somalia, South Sudan, Sudan and Uganda) and over 80 million people are already struggling to feed themselves and their families.

WHO announced that it was scaling up operations in the region to ensure access to essential health services. These include treating children with severe malnutrition and responding to infectious disease outbreaks. WHO is also working with local authorities on disease surveillance systems to ensure timely outbreak detection.

https://bit.ly/3yHXPIX

## Afghanistan earthquake response

An earthquake hit Afghanistan on 22 June, devastating the provinces of Paktika and Khost in the south-east of the country. Initial reports stated that more than 1000 people had been killed and some 3000 injured. As of 28 June, the health facilities that remained operational in the area were reporting shortages of surgical medicines and supplies to treat the injured.

Immediately following the earthquake, WHO sent medical supplies from its warehouse in Kabul to health facilities in the affected provinces, including enough trauma supplies for 5400 surgeries and medical treatments for 36 000 patients for three months. WHO mobile health teams and ambulances were also sent.

On 28 June, a flight containing 25 metric tonnes of WHO health supplies landed in Kabul from WHO’s logistics hub in Dubai. As of that same date, six hospitals supported by WHO were operating at full capacity in the affected area, providing trauma care and physical rehabilitation.

https://bit.ly/3NRdN82

## Ebola outbreak ends

On 4 July the Democratic Republic of the Congo declared the end of the Ebola outbreak that started less than three months earlier. The swiftness of the response to the outbreak was credited for its early conclusion.

National emergency teams, with support from WHO and partners, responded quickly after the outbreak was declared on 23 April, implementing measures that included testing, contact tracing, infection prevention and control, treatment and community engagement.

A focused vaccination campaign was launched just four days after the outbreak was declared, reaching 2104 people, including 302 contacts and 1307 frontline health workers. In all, there were four confirmed infections and one probable infection. All five people died. It was the third outbreak in the province since 2018 and the country’s 14th overall.

https://bit.ly/3bThRHG

## First Marburg virus in Ghana

Preliminary analysis of samples taken from two patients in Ghana indicated the presence of Marburg virus. As of 7 July, the samples had been sent to the Institut Pasteur in Senegal, for confirmation. If confirmed, these would the first Marburg infections recorded in the country.

WHO deployed experts to support Ghana’s health authorities by bolstering disease surveillance, testing, contact tracing, preparing to treat patients and working with communities to alert and educate them about the risks and dangers of the disease and to collaborate with the emergency response teams.

In the same family as Ebola virus, Marburg is likewise associated with haemorrhagic fever and a high case fatality rate.

https://bit.ly/3ABarDg

## Monkeypox spreading

Monkeypox continued to spread, with over 80% of reported cases in the WHO European Region. As of 4 July 2022, 6027 laboratory confirmed cases of monkeypox and three deaths had been reported to WHO since 13 May, with cases being reported in 59 countries/territories/areas. Some 2614 cases were reported between 27 June and 4 July.

The overall risk of further spread is assessed to be moderate at global level but high in the European Region due to reports of a widespread outbreak involving several countries.

WHO continues to closely monitor the situation, and support international coordination and information sharing with Member States and partners. Clinical and public health incident responses have been activated by Member States to coordinate comprehensive case finding, contact tracing, laboratory investigation, isolation, clinical management and implementation of infection and prevention and control measures.

https://bit.ly/3Pg3pIe

https://bit.ly/3yi6xwc

## Hepatitis of unknown cause

WHO launched a global online survey to assess whether there has been an increase in incidence of severe acute hepatitis of unknown etiology in children ≤16 years relative to the period 2017–2021. The survey will also seek to identify any changes in the age distribution and severity of reported cases. Launched on 11 July, the voluntary survey was shared across networks of paediatric hepatologists and other specialist paediatricians, requesting aggregated data.

Between 5 April (when the outbreak was initially detected) and 8 July 2022, 35 countries in five WHO regions had reported 1010 probable cases and 22 deaths. Almost half (48%) of the probable cases were reported from the WHO European Region, including 272 cases (27% of global cases) from the United Kingdom of Great Britain and Northern Ireland.

As of 12 July, the mode of transmission and the etiologic agent(s) had not been determined.

https://bit.ly/3uNesk3

## Funding the ACT-Accelerator 

The governments of Norway and Sweden made contributions of US$ 340 million and US$ 300 million respectively to the ACT-Accelerator, the international funding mechanism established to ensure equitable access to COVID-19 vaccines, treatments and technologies. In doing so, they joined Germany in having exceeded their “fair share” for the ACT-Accelerator’s 2021/22 budget. The ACT-Accelerator now faces a funding gap of US$ 11.2 billion, having received contributions totalling US$ 5.6 billion for the 2021/22 budget.

In a 4 July statement, WHO Director-General Tedros Adhanom Ghebreyesus commended the countries for their contributions and called on others to follow their lead, noting that the pandemic is far from over.

He returned to the point in a 12 July media briefing in which he highlighted challenges that include the spread of Omicron sub-variants, a widespread reduction in testing and sequencing and ineffective deployment of diagnostics, treatments and vaccines. 

https://bit.ly/3bDYdiz

https://bit.ly/3yBanAK

## Antibiotic pipeline "stagnant"

Not enough new antibacterial treatments are being developed to address the mounting threat of antibiotic resistance. This is according to WHO’s annual antibiotic pipeline report published on 22 June.

The 2021 report describes the antibacterial clinical and preclinical pipeline as stagnant, noting that since 2017 only 12 antibiotics have been approved, 10 of which belong to existing classes with established mechanisms of antimicrobial resistance. In 2021 only 27 new antibiotics were in clinical development against priority pathogens, down from 31 products in 2017.

https://bit.ly/3yHXCWo

## Vaccine candidates for antimicrobial-resistant pathogens

WHO released the inaugural report on the pipeline of vaccines intended to prevent infections caused by antimicrobial-resistant (AMR) bacterial pathogens on WHO’s bacterial priority pathogens list.

Published on 12 July, the report identifies 61 vaccine candidates in different stages of clinical development but notes that even those in late-stage development are unlikely to be available in the short term. The report calls for the acceleration of late-stage trials for AMR-related vaccines, leveraging of vaccine technologies that have come to the fore in tackling COVID-19, and full, optimized use of existing vaccines.

https://bit.ly/3PpACB2

**Cover photo**

Community Health worker, Tambudzai Vumisai, collects her UNICEF-sponsored bicycle at her house in Nyahode, Zimbabwe, 6 February 2020.

**Figure:**
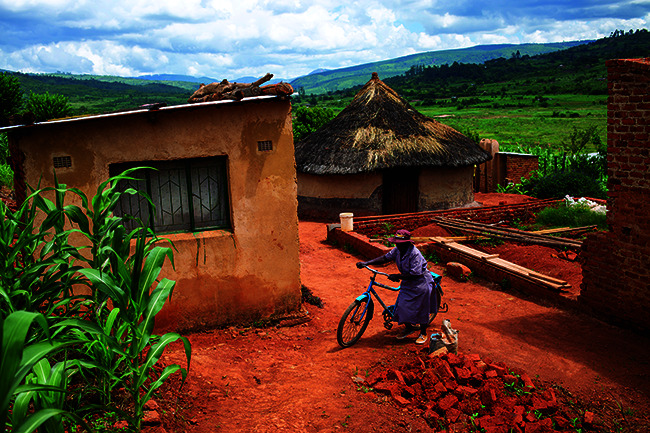
UNICEF/UNI296619/Prinsloo

## Accelerating access to genomics

WHO’s Science Council issued its first report on accelerating access to genomics for global health. Published 12 July, the report calls for expanding access to genomic technologies, particularly in low- and middle-income countries, by addressing shortfalls in financing, laboratory infrastructure, materials and highly trained personnel.

https://bit.ly/3NWYpHi

## Ultraviolet radiation app

A new app for mobile phones that provides localized information on ultraviolet (UV) radiation levels was launched by WHO and partners on 21 June. The SunSmart Global UV app is designed to let people know when to use skin and eye protection in an effort to reduce the global burden of skin cancer as well as UV-related eye damage.

https://bit.ly/3NJrkyt

**Looking ahead**

1–7 August, World Breastfeeding Week. https://bit.ly/3yklpu3

31 August–2 September, Health-enhancing physical activity (HEPA) Europe 2022 Conference. https://bit.ly/3tEGL3H

1–2 September, H20: Annual meeting of the G20 health ministers’ group. https://bit.ly/3uE39L6

